# Epidemiology of Adenovirus Pneumonia and Risk Factors for Bronchiolitis Obliterans in Children During an Outbreak in Jilin, China

**DOI:** 10.3389/fped.2021.722885

**Published:** 2021-09-28

**Authors:** Xiuhua Yu, Yucong Ma, Yang Gao, Hailong You

**Affiliations:** Department of Pediatrics, First Hospital of Jilin University, Changchun, China

**Keywords:** adenovirus pneumonia, post-infectious bronchiolitis obliterans, epidemiology, risk factors, children

## Abstract

**Background:** Jilin Province, located in northeastern China, recently experienced a human adenovirus (HAdV) epidemic. Few studies involving hospitalized pediatric patients with pneumonia caused by HAdV in our region exist. HAdV pneumonia can lead to severe long-term respiratory sequelae, such as post-infectious bronchiolitis obliterans (PIBO), which has a poor prognosis and greatly influences the quality of life of pediatric patients. However, studies on the risk factors for PIBO are limited.

**Objective:** To describe the HAdV pneumonia prevalence and determine potential risk factors for PIBO development among hospitalized children in Jilin Province, China.

**Methods:** The data of 187 children with HAdV pneumonia (10 months−12 years old) admitted to the First Hospital of Jilin University during an outbreak between October 2018 and January 2020 were retrospectively studied. We analyzed the epidemiological characteristics of HAdV pneumonia, focusing on severe HAdV pneumonia (66 cases). The risk factors for BO development were determined by comparing the demographic and clinical data of the BO and non-BO groups.

**Results:** The largest number of HAdV pneumonia cases occurred in January 2019 (severe *n* = 18, general *n* = 21), followed by December 2018 (severe *n* = 14, general *n* = 11), June 2019 (general *n* = 17), July 2019 (general, *n* = 14), and May 2019 (general, *n* = 13). In total, 91.98% of the children with HAdV pneumonia were <6 years old (172/187), and 50% of the pediatric patients with severe HAdV pneumonia were <2 years old (33/66). We found that 30.3% of the severe cohort developed BO (20/66), and the strongest independent risk factors for PIBO were persistent wheezing (OR 181.776, 95% CI, 3.385–9,761.543) and acute respiratory failure (OR 51.288, 95% CI, 1.858–1,415.441) during a severe pneumonia episode.

**Conclusions:** The largest number of HAdV pneumonia cases, especially severe cases, occurred in winter in Northeast China, followed by summer. The majority of children admitted with HAdV pneumonia were <6 years old, and half of severe HAdV pneumonia patients were <2 years old. Children who had persistent wheezing or acute respiratory failure during the acute phase of severe HAdV pneumonia were prone to the development of BO.

## Introduction

Human adenovirus (HAdV) infection is associated with a wide spectrum of disease, including conjunctivitis, gastroenteritis and respiratory tract infection. This pathogen is prevalent in children with acute low respiratory tract infection (ALRTI) and accounts for ~4–10% of all cases of childhood pneumonia (1). Although HAdV pneumonia cases are often mild to moderate in severity, HAdV pneumonia can be serious or even fatal and can cause long-term respiratory complications such as bronchiectasis and post-infectious bronchiolitis obliterans (PIBO) (2), especially in younger children (3).

It is well-recognized that the incidence of PIBO has increased in some South American and Asian countries (Argentina, Chile, Brazil, South Korea, etc.) (4–7), including mainland China (8, 9). BO is uncommon and characterized by severe small airway injury–related airflow obstruction. Infection, bone marrow transplantation, connective tissue diseases, and exposure to toxic gases or certain drugs may lead to the development of BO (10). Concentric narrowing and obliteration of the bronchiolar lumen are characteristic of BO (11). In children, PIBO is most common form of BO. In a number of infections, HAdV is by far most often associated with PIBO (12, 13). Histopathological confirmation by lung biopsy is a definitive method for the diagnosis of BO, but it is invasive and does not always provide evidence because of the patchy distribution of lesions (14). PIBO can be diagnosed based on typical clinical manifestations and characteristics on high-resolution computed tomography (HRCT), including mosaic perfusion patterns and/or bronchiectasis (13, 15). Examination of the pulmonary function of these patients usually demonstrates severe and fixed airway obstruction (16). Although the severity of the disease varies, the overall prognosis and quality of life in pediatric patients are poor (16). Additionally, there is no widely accepted protocol for treatment. Thus, identifying HAdV pneumonia patients at risk of developing BO and initiating early interventions are very important. However, studies focusing on the risk factors for BO after severe HAdV pneumonia are limited.

The epidemic characteristics of HAdV vary among different countries and regions as well as different time periods (17). Jilin Province, located in northeastern China, has experienced several epidemic outbreaks of HAdV in recent years. There are few studies focusing on hospitalized pediatric patients with HAdV pneumonia in our region. This research aims to describe the prevalence of pneumonia caused by HAdV and determine the potential risk factors for PIBO development among children hospitalized in Jilin Province, China.

## Methods

### Selection of Patients

The data of 187 children who contracted HAdV pneumonia during an outbreak between October 2018 and January 2020 and were admitted to the pediatric respiratory department of the First Hospital of Jilin University were retrospectively analyzed. The included patients were aged between 10 months and 12 years old. The patients were followed up until May 2021.

HAdV pneumonia was diagnosed based on clinical manifestations, radiological confirmation (made by a pediatric radiologist), and a positive nasopharyngeal secretion adenovirus antigen immunofluorescence result. Severe pneumonia was determined according to The Chinese Medical Association Guidelines for the diagnosis and treatment of community-acquired pneumonia (CAP) in children as shown in Table 1 (18). Any included HAdV pneumonia patient with the presence of any one of the severe conditions was considered to have severe HAdV pneumonia.

**Table 1:** Assessment of CAP severity in children.

**Item**	**General**	**Severe**
General condition	Well	Poor
Disordered consciousness	No	Yes
Hypoxemia	No	CyanosisRespiratory rate significantly increased (≥70 times/min for infants, ≥50 times/min for children)Dyspnea (moans, nasal flaring, retractions)Intermittent apnea
Fever	Failure to meet severe criteria	Very high fever or continuous high fever for more than 5 days
X-ray or CT scan	Failure to meet severe criteria	Unilateral pulmonary infiltration ≥ 2/3 or multilobe involvementPneumothoraxPleural effusionPulmonary abscessPulmonary necrosis
Extrapulmonary complications	No	Yes
Criteria	With the presence of all the above conditions	With the presence of any one of the above conditions

*CAP, community-acquired pneumonia*.

The severe HAdV pneumonia patients were divided into two groups according to the development of PIBO during follow-up: the BO group and the non-BO group. For the diagnosis of PIBO, the following five criteria should be met: a. persistent cough, wheezing and tachypnea accompanied by varying degrees of dyspnea and decreased activity tolerance for more than 6 weeks after an acute severe HAdV pneumonia event; b. extensive wheezing and moist crackles in the lungs on auscultation; c. mosaic perfusion patterns, bronchial wall thickening, and/or bronchial dilation on chest HRCT; d. airway obstruction on pulmonary function evaluation; and e. exclusion of diagnoses of asthma, bronchopulmonary dysplasia, cystic fibrosis, and pulmonary tuberculosis.

In addition, patients with an organ or bone marrow transplantation history, with a connective tissue disease, who were admitted to the hospital during the recovery period, and who had severe pneumonia cause by another pathogen before the diagnosis of BO after discharge were excluded.

The study was approved by the research ethics committees of the institutions. All legal guardians of the participants consented to inclusion in the study.

### Data Collection

Data were obtained from the electronic clinical record system of our hospital. We analyzed the epidemiological characteristics of HAdV pneumonia during the outbreak, focusing on the characteristics of patients with severe HAdV pneumonia, including demographics, significant comorbidities, clinical manifestations, laboratory and imaging findings, coinfection, extrapulmonary complications and primary treatments.

Influenza A/B virus, respiratory syncytial virus, and parainfluenza virus antigen were determined by nasopharyngeal secretion immunofluorescence tests. *Mycoplasma pneumoniae* and *Chlamydia pneumoniae* were identified by polymerase chain reaction. Sputum and blood culture were performed to detect bacterial pathogens. Due to financial and clinical limitations in our hospital, adenovirus serotype analysis was not performed.

All PIBO patients underwent HRCT (SOMATOM Sensation Cardiac 64 CT scanner, Siemens AG, Forchheim, Germany) and pulmonary function testing (Master Screen Paed, Jaeger Company, Wurzburg, Germany) at the time of diagnosis. Airway obstruction was defined as a time to reach peak tidal expiratory flow as a proportion of total expiratory time (tPTEF%tE) <30% and a ratio of volume to reach peak expiratory flow to total expiratory volume (VPEF%VE) <30% (mild, 23–28%; moderate, 15–23%; severe, <15%) of tidal breathing pulmonary function (patients aged <3 years) or Z5 (respiratory system impedance at 5 Hz) and R5 (resistance of respiratory system at 5 Hz) > 120% of the predicted value on impulse oscillometry (patients aged 3–6 years).

### Statistical Analysis

SPSS 26.0 and R 3.5.3 software were used for statistical analysis, and the Shapiro-Wilk method was used to test for normality. Normally distributed quantitative data are expressed as means(SD), and the independent sample *t*-test was used for comparisons between groups. Non-normally distributed quantitative data are expressed as medians and interquartile ranges (IQRs), and comparisons between groups were performed with the rank sum test. Qualitative data are described as frequencies (composition ratios), and differences between groups were calculated by the chi-square test. Logistic regression was used to analyze each factor. Considering the possibility of overfitting, we used lasso regression to eliminate variables with a β value of 0. Variables with a *p*-value < 0.05 were included in the multivariate logistic regression analysis. A *p*-value < 0.05 was considered to be statistically significant.

## Results

### Epidemiology of HAdV Pneumonia

**Figure 1:**
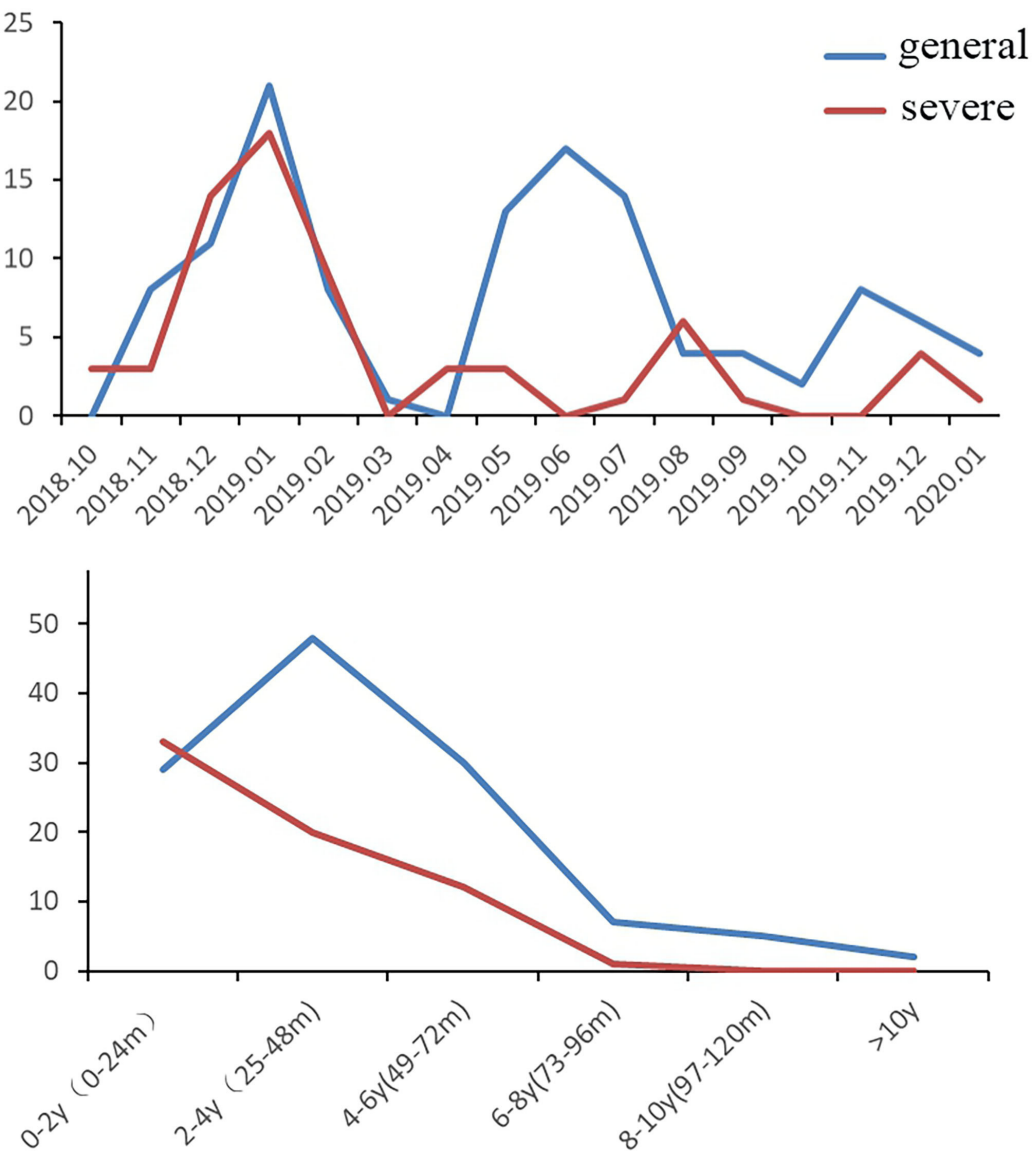
Trends of hospitalized patients with HAdV pneumonia (*n* = 187, general cases = 121, severe cases = 66).

During the HAdV outbreak from October 2018-January 2020, 187 patients were hospitalized for HAdV pneumonia in our department, of whom 66 (35.3%) had severe HAdV pneumonia. Figure 1 shows the trends of hospitalized patients with HAdV pneumonia (severe and general cases) during the study period. The largest number of HAdV pneumonia cases occurred in January 2019 (severe *n* = 18, general *n* = 21) and December 2018 (severe *n* = 14, general *n* = 11), followed by June 2019 (general *n* = 17), July 2019 (general, *n* = 14), and May 2019 (general, *n* = 13); these values correspond to the 2 highest peaks in Figure 1. Regarding age, half of the pediatric patients with severe HAdV pneumonia were <2 years old (*n* = 33), followed by 2–4 years old (*n* = 20) and 4–6 years old (*n* = 12). General cases were mainly distributed in 0- to 2-year-olds (*n* = 29), 2- to 4-year-olds (*n* = 48), and 4- to 6-year-olds (*n* = 30). In summary, 98.5% of the children with severe HAdV pneumonia and 88.4% of those with general HAdV pneumonia were <6 years old, for an overall total of 91.98%.

### Characteristics of Children With Severe HAdV Pneumonia

**Figure 2:**
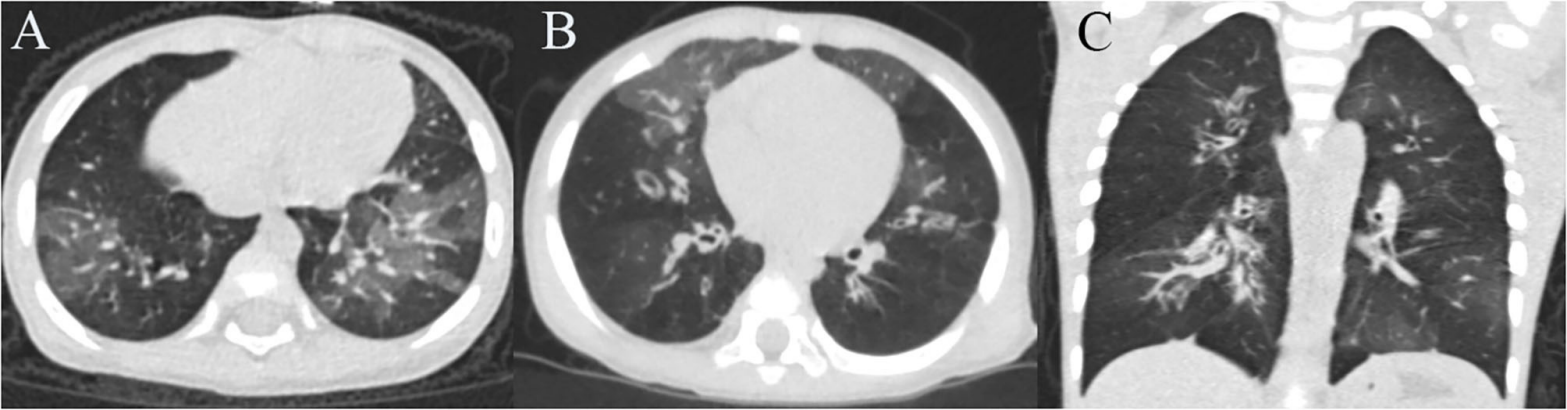
Chest HRCT scans of a 16-month-old girl and a 8-month-old boy with PIBO. **(A)** Mosaic perfusion pattern and bronchial wall thickening of the girl. **(B)** Mosaic perfusion pattern, bronchial wall thickening and bronchiectasis of the boy **(C)** 3D reconstruction (the boy).

Of the 66 included patients with severe HAdV pneumonia (male/female ratio 1.28:1), 20 (30.3%) developed BO during follow-up. At the time of diagnosis, all these PIBO patients had mosaic perfusion patterns and bronchial wall thickening on HRCT, and eight children presented bronchiectasis (Figure 2). In terms of pulmonary function, nineteen children showed severe airway obstruction on tidal breathing pulmonary function testing, and one patient showed significantly increased Z5 and R5 values. Throughout the follow-up period (after the acute severe HAdV pneumonia event, the median follow-up time of these PIBO patients was 27 months), the HRCT findings were persistent, and the symptoms could not be completely resolved even after treatment. A summary of the demographic characteristics and clinical features of the patients in the two groups (children who developed BO and those who did not) is shown in Table 2. The median age in the BO groups was 16.5 (IQR: 11–25.25 months) months, while that in the non-BO group was 30.5 (IQR: 17–50.75 months) months; and there was a statistically significant difference (*P* < 0.05). No significant difference was found in sex or ethnicity between the two groups.

**Table 2:** Comparisons of demographic and clinical characteristics of pediatric patients in the BO and non-BO groups.

	**BO group** (***n*** **= 20)**	**Non-BO group** (***n*** **= 46)**	***P***
Age in months, median (IQR)	16.50 (11.00, 25.25)	30.50 (17.00, 50.75)	0.005
Male, *n* (%)	13 (65.0)	24 (52.2)	0.487
Ethnicity C:M:O ratio, *n*	18:2:0	43:2:1	0.552
**Significant comorbidities**, ***n*** **(%)**			
Premature birth	2 (10.0)	3 (6.5)	0.401
Congenital heart disease	0 (0.0)	1 (2.2)	1.000
A history of wheezing	3 (15.0)	2 (4.3)	0.148
Family asthma history	1 (5.0)	0 (0.0)	0.666
Others	1 (5.0)	2 (4.3)	1.000
**Clinical manifestations**			
Length of hospital stay, d (median, IQR)	21.50 (14.50, 29.25)	13.00 (10.25, 18.75)	0.005
Duration of fever, d (median, IQR)	15.50 (10.75, 21.50)	10.00 (8.00, 15.00)	0.007
Peak temperature, °C (mean (SD))	39.76 (0.47)	39.76 (0.49)	0.975
Duration of cough, d (median, IQR)	38.50 (30.00, 52.25)	26.00 (18.00, 30.00)	<0.001
Persistent wheezing, *n* (%)	19 (95.0)	13(28.3)	<0.001
Dyspnea, *n* (%)	18 (90.0)	16(34.8)	<0.001
Hypoxemia, *n* (%)	18 (90.0)	13(28.3)	<0.001
Hypercapnia, *n* (%)	7 (35.0)	0(0.0)	<0.001
Respiratory failure, *n* (%)	14 (70.0)	8(17.4)	<0.001
**Laboratory findings**			
WBC (× 10^9^/L) (median, IQR)	9.82 (3.83, 14.99)	7.04 (4.81, 10.88)	0.418
HGB, g/l (mean (SD)	97.90 (16.34)	110.51 (14.05)	0.002
PLT (×10^9^/L) (median, IQR)	335.50 (200.75, 406.00)	260.50 (169.25, 366.00)	0.244
Neutrophil proportion,% (median, IQR)	71.00 (58.00, 81.50)	58.50 (47.25, 73.75)	0.026
Lymphocytes proportion,% (median, IQR)	21.00 (14.00, 33.75)	34.50 (21.50, 44.75)	0.038
CRP, mg/l (median, IQR)	21.13 (8.51, 37.54)	17.91 (9.20, 34.84)	0.967
CK-MB, U/L (median, IQR)	36.05 (30.10, 51.75)	29.50 (24.52, 35.90)	0.008
LDH, U/L (median, IQR)	898.50 (582.50, 1325.00)	613.00 (410.00, 940.75)	0.060
AST, U/L (median, IQR)	64.60 (44.17, 93.25)	51.10 (41.02, 82.55)	0.295
ALT, U/L (median, IQR)	16.05 (12.95, 24.15)	18.55 (11.35, 26.82)	0.796
**Imaging findings**, ***n*** **(%)**			
Segmental pulmonary consolidation	3 (15.0)	10 (21.7)	0.767
Atelectasis	5 (25.0)	13(28.3)	1.000
Pleural effusion	3 (15.0)	16(34.8)	0.251
**Co-infection**, ***n*** **(%)**			
MP/CP	4 (20.0)	13 (28.3)	0.107
Bacteria	5 (25.0)	12 (26.1)	1.000
Other virus	5 (25.0)	10 (21.7)	1.000
**Extrapulmonary complications**, ***n*** **(%)**			
Nervous system	0 (0.0)	2 (4.3)	0.868
Digestive system	8 (40.0)	19 (41.3)	1.000
**Treatment**			
Invasive mechanical ventilation, *n* (%)	10 (50.0)	3 (6.5)	<0.001
Length of mechanical ventilation, d (median, IQR)	8.00 (7.00, 9.75)	9.00 (8.00, 11.00)	0.670
Use of CPAP, *n* (%)	5 (25.0)	4 (8.7)	0.127
Use of γ-globulin, *n* (%)	17 (85.0)	33 (71.7)	0.399
Use of glucocorticoids, *n* (%)	20 (100.0)	46 (100.0)	1.000
Duration of glucocorticoid use, d (median, IQR)	8.50 (6.75, 15.25)	7.50 (6.00, 10.00)	0.105

*Data are shown as the mean (SD), median (IQR, interquartile range) or number (n, %). BO, bronchiolitis obliterans; C:M:O: Chinese:Mongolian:Other; WBC, white blood cell; HGB, hemoglobin; PLT, platelet; CRP, C-reaction protein; CK-MB, creatine kinase-MB; LDH, lactate dehydrogenase; AST, aspartate aminotransferase; ALT, alanine aminotransferase; ALB, albumin; MP, mycoplasma; CP, chlamydia; CPAP, continuous positive airway pressure*.

In total, 15 patients (22.7%) had comorbidities of prematurity (*n* = 5, 7.6%), congenital heart disease (*n* = 1, 1.5%), a history of wheezing (*n* = 5, 7.6%), or a family history of asthma (*n* = 1, 1.5%). In other words, most of the included patients were healthy before developing severe HAdV pneumonia. Regarding clinical characteristics, during the acute phase of severe HAdV pneumonia, 48.5% of patients had wheezing, 51.5% had dyspnea, 46.97% had hypoxemia, 10.6% had hypercapnia, and 33.3% had respiratory failure. Segmental pulmonary consolidation was observed in 19.7% of the patients, atelectasis in 27.3%, and pleural effusion in 28.8%. Among the patients, digestive system conditions, including vomiting, diarrhea, and damaged liver function, were the primary extrapulmonary manifestations (40.9%). In addition, only two cases had meningitis and toxic encephalopathy, respectively. Coinfection was common; the rates of mixed *M. pneumoniae* and *C. pneumoniae* infection, mixed bacterial infection, and other virus infection were 25.8, 25.8, and 22.7%.

The patients in the BO group had significantly longer hospitalizations; had longer durations of fever and cough; were more likely to experience persistent wheezing, dyspnea, hypoxemia, hypercapnia, and respiratory failure; and were more likely to require invasive mechanical ventilation than those in the non-BO group (*P* < 0.05). Laboratory findings revealed that children with BO had a lower lymphocyte percentage and hemoglobin level and a higher neutrophil proportion and creatinine kinase (CK)-MB level than those without BO (*P* < 0.05).

### Risk Factors for BO in Children With Severe HAdV Pneumonia

Table 3 shows the potential risk factors for the development of BO in the univariate and multivariate analyses. Eleven variables with statistical significance were selected after univariate analysis. Multivariate analysis showed that the strongest independent risk factors for BO were persistent wheezing [odds ratio (OR) 181.776, 95% confidence interval (CI) 3.385–9,761.543, *P* < 0.05] and acute respiratory failure (OR 51.288, 95% CI 1.858–1,415.441, *P* < 0.05) in the acute phase of severe HADV pneumonia.

**Table 3:** Logistic regression analysis of potential independent risk factors for postadenovirus BO.

	**Univariate analysis**		**Multivariate analysis**	
	**OR (95% CI)**	***p***	**OR (95% CI)**	***p***
Persistent wheezing	53.833 (6.488, 446.642)	0.000	181.776 (3.385, 9761.543)	0.010
Respiratory failure	16.714 (4.587, 60.898)	0.000	51.288 (1.858, 1415.441)	0.020
Age in months	1.097 (1.028, 1.17)	0.005		
Length of hospital stay, d	0.944 (0.906, 0.985)	0.007		
Duration of fever, d	1.125 (1.033, 1.226)	0.007		
Duration of cough, d	1.095 (1.04, 1.153)	0.001		
Dyspnea	10.625 (2.702, 41.779)	0.001		
Hypoxemia	22.846 (4.633, 112.666)	0.000		
HGB	0.946 (0.91, 0.983)	0.005		
CK-MB	1.048 (1.01, 1.089)	0.014		
Invasive mechanical ventilation	14.333 (3.321, 61.858)	0.000		

*BO, bronchiolitis obliterans; OR, odds ratio; CI, confidence interval; HGB, hemoglobin; CK-MB, creatine kinase-MB*.

A receiver operating characteristic (ROC) curve was constructed to assess the predictive value of the model. The area under the curve was 0.934 (95% CI 0.878–0.991, *P* < 0.05), indicating diagnostic accuracy. The cutoff value was 0.052, with a sensitivity of 1.000 and specificity of 0.717.

## Discussion

This study provides insights into the trends of HAdV pneumonia in Northeast China between 2018 and 2020, particularly regarding potential risk factors for the development of PIBO in patients with severe HAdV pneumonia. We found that HAdV pneumonia generally occurred year-round in Northeast China, but it was most common in winter, especially severe HAdV pneumonia, followed by summer. In our study, the majority of pediatric patients were <6 years old (91.98%), similar to previous studies (19–21). Liu et al. found that most patients with HAdV lower respiratory tract infection (LRTI) were <5 years old (88.35%) (22). Among those with severe cases, half were <2 years old. This is similar to findings from Singapore, where most children with severe HAdV were <2 years old (23, 24). It has been reported that “young children are at increased risk of severe HAdV infection” (25). The reason may be that the immune systems of younger children, especially those <2 years of age, are very immature, resulting in more severe HAdV infection. However, age was not an independent risk factor for respiratory complications after HAdV infection.

Previous research has shown that 14–60% of HAdV LRTIs result in sequelae, such as BO, bronchiectasis, and fibrosis, with varying degrees of severity (26). In our cohort, 30.3% (20/66) of the children with severe HAdV pneumonia developed BO, similar to that in some previous studies (4, 27). The patients in the BO group had significantly longer hospitalization durations; had longer of fever and cough durations; were more likely to experience persistent wheezing, dyspnea, hypoxemia, hypercapnia, respiratory failure; and were more likely to require invasive mechanical ventilation. A 5-year follow-up study of 38 HAdV pneumonia patients showed that children who developed BO had significantly more severe respiratory compromise [higher rates of accessory muscle use, crackles, intensive care unit (ICU) admission, and mechanical ventilation; a longer duration of oxygen therapy; and more medication use] and a longer duration of hospitalization than those who did not (5). The development of BO was associated with acute insult severity, and it seemed that the more severe the episode of HAdV pneumonia was, the higher the incidence of BO was.

Accordingly, analysis of predictors of PIBO showed that factors representing acute illness severity were correlated with BO development. In our study, acute respiratory failure during the acute severe HAdV pneumonia stage was an independent risk factor for PIBO. A retrospective observational study in 415 hospitalized children with acute low respiratory infection caused by HAdV found that the independent risk factors for BO development were >30 days of hospitalization, multifocal pneumonia and hypercapnia (4). Zhong et al. observed that fever durations longer than 10.5 days, dyspnea and mechanical ventilation were indicators of PIBO in a case-control study in 139 patients with severe adenovirus pneumonia (27). In a study in Southeast China, Wu et al. considered hypoxemia to be the only predictor of BO following respiratory adenoviral infection in children (28). A study in Kuala Lumpur, Malaysia, showed that the need for non-invasive ventilation (NIV) and pediatric intensive care unit (PICU) admission were independent risk factors for respiratory complications after HAdV infection (29). Indeed, almost all of our patients who had respiratory failure were admitted to the PICU and received NIV or invasive ventilation (IV). Although longer durations of hospitalization and fever, dyspnea and hypoxemia were not independent factors, they were potential risk factors for PIBO in the univariate logistic regression analyses in our study. These results are similar to ours, suggesting that the risk of PIBO is increased in pediatric patients suffering from severe respiratory compromise caused by HAdV.

Persistent wheezing during a severe HAdV pneumonia episode was another independent risk factor for PIBO in our investigation. The histopathological basis of wheezing is airway narrowing as a result of inflammation, which can cause swelling, congestion, excess secretion, and scarring. Damage and repair of the bronchioles may lead to narrowing and obliteration of the lumen, which is characteristic of BO. Thus, we considered persistent wheezing to be a predictor of BO, as persistent wheezing may reflect injury to the small airways caused by HAdV and subsequent inflammatory processes.

The roles of host factors in the development of PIBO should be given more attention. A family asthma history and previous wheezing episodes were found to be associated with the development of PIBO in some studies (5, 29), but in our study, there was no significant difference between the BO group and non-BO group. The published risk factors are not totally consistent with ours, which may be due to the dissimilar populations, issues related to different of inclusion and exclusion criteria or the variables analyzed. In addition, coinfection with bacteria, other viruses or *M. pneumoniae* did not increase the risk of respiratory complications, and radiology was not predictive of the development of BO.

Our study has several limitations. First, the HAdV serotype could not be determined at our hospital, and Ad7 can cause severe lung damage and lead to BO or even death. Second, the sample size was not large. Finally, this was a single-center study.

In summary, during the dramatic increase in the number of HAdV infections in Jilin Province, China, between October 2018 and January 2020, the largest number of HAdV pneumonia cases, especially severe cases, occurred in winter, followed by summer. The majority of pediatric patients admitted for HAdV pneumonia were aged <6 years, while half of severe HAdV pneumonia patients were aged <2 years. In total, 30.3% of the severe cohort developed BO. The presence of persistent wheezing and acute respiratory failure during a severe HADV pneumonia episode were independent risk factors for the development of PIBO. Further investigations are needed to determine the mechanisms by which HAdV causes BO.

## Data Availability Statement

The original contributions presented in the study are included in the article/supplementary material, further inquiries can be directed to the corresponding author/s.

## Ethics Statement

The studies involving human participants were reviewed and approved by the research Ethics Committees of First Hospital of Jilin University. Written informed consent to participate in this study was provided by the participants' legal guardian/next of kin.

## Author Contributions

XY undertook the follow-up of patients, data collection, analysis, and produced the manuscript. YM was responsible for the follow-up of patients and data collection. YG undertook data analysis and a literature review. HY was for the whole article and financial support as corresponding author. All authors contributed to the article and approved the submitted version.

## Funding

This study was supported by the 11th Youth Development Fund of First Hospital of Jilin University (JDYY11202017).

## Conflict of Interest

The authors declare that the research was conducted in the absence of any commercial or financial relationships that could be construed as a potential conflict of interest.

## Publisher's Note

All claims expressed in this article are solely those of the authors and do not necessarily represent those of their affiliated organizations, or those of the publisher, the editors and the reviewers. Any product that may be evaluated in this article, or claim that may be made by its manufacturer, is not guaranteed or endorsed by the publisher.
